# Spiritually and Religiously Integrated Group Psychotherapy: A Systematic Literature Review

**DOI:** 10.1155/2013/274625

**Published:** 2013-10-31

**Authors:** Dorte Toudal Viftrup, Niels Christian Hvidt, Niels Buus

**Affiliations:** ^1^Health, Man and Society, Institute of Public Health, SDU, Odense J. B. Winsløwsvej 9B, 5000 Odense C, Denmark; ^2^Clinic and Policlinic for Palliative Medicine, LMU, Marchioninistraße 15, 1377 Munich, Germany; ^3^Freiburg Institute for Advanced Studies (FRIAS 2012-14), Stadtstraße 5, 79104, Germany

## Abstract

We systematically reviewed the research literature on spiritually and religiously integrated group psychotherapy to answer the following three questions: first, how are spirituality and religiosity defined; second, how are spiritual and religious factors characterized and integrated into group psychotherapy; and, third, what is the outcome of the group psychotherapies? We searched in two databases: PsycINFO and PubMed. Inclusion and exclusion criteria and checklists from standardized assessment tools were applied to the research literature. Qualitative and quantitative papers were included. In total, 8 articles were considered eligible for the review. Findings from the evaluation suggested that the concepts of spirituality and religiosity were poorly conceptualized and the way in which spiritual and religious factors were integrated into such group psychotherapies, which distinguished it from other types of group psychotherapies, was not fully conceptualized or understood either. However, clear and delimited conceptualization of spiritual and religious factors is crucial in order to be able to conclude the direct influences of spiritual or religious factors on outcomes. Implications for spiritually or religiously integrated group psychotherapy and conducting research in this field are propounded.

## 1. Introduction 

Spirituality and religion have received increased attention in health research, and they appear to be mostly associated with quality of life and improved health [1, 2]. The role of spirituality and religiosity in physical and mental health has been addressed in medical, psychiatric, psychological, and behavioral medicine journals, and evidence suggests links between improved health and spirituality and religiosity [3]. For example, a Danish cohort study with 10800 Baptists and Adventists has pointed to decreased risk of cancer, COLD, coronary heart disease, and some psychiatric disorders. [4]. Moreover, spirituality and religion have also been increasingly viewed as important components of people's lives that can be successfully attended to in mental health treatment [5]. Several studies indicate that spiritual and religious people benefit from spiritually and religiously integrated interventions [5], and there is a substantive body of literature on how to integrate spirituality and religion into psychotherapy [6, 7]. For example, Rye et al. [8] investigated the effectiveness of secular and religious forgiveness interventions. However, they found no significant differences when directly comparing secular and religious participants on primary or secondary outcomes. Different therapeutic approaches with an integration of spirituality and religiosity [3, 9] and psychotherapy with specific religious groups [10] have been propounded. However, the integration of spiritual and religious factors is not fully understood. Until now, most empirical studies on spiritually and religiously integrated psychotherapy have evaluated the effectiveness of the complete intervention, but a clarification of the spiritual and religious factors, separating spiritually and religiously integrated psychotherapy from other types of group psychotherapy, remains unanswered.

Furthermore, integration of spirituality and religion into group psychotherapy is an underresearched area of inquiry compared to psychotherapy with individuals [6, 7, 11]. The relatively few empirical studies on spiritually and religiously integrated group psychotherapy focus on the effectiveness of the complete intervention [5]. However, the way in which these studies integrate spiritual and religious factors into group psychotherapy and what constitutes these effects remain unclear.

The paucity of studies on group intervention with integration of spirituality and religion is surprising because spirituality and religion most often develop and are practiced in communities with groups of people who share the same convictions and understandings and because religion is a group phenomenon, one of the earliest forms of a large group [12]. Psychological group interventions, which integrate spirituality and religion, might therefore benefit more from the psychological dynamics of spirituality and religion than individual interventions. Studies indicate that group psychotherapy interventions are time efficient, economical, and effective in improving coping skills and quality of life and reducing psychological and physical distress [13, 14].

More research-based knowledge about the spiritual and religious factors and the effects they have in spiritually and religiously integrated group psychotherapy may be beneficial to healthcare. We therefore undertook a systematic search of the literature to explore studies on spiritually and religiously integrated group psychotherapy. The purpose was to critically evaluate and summarize state of knowledge concerning the complexity of spiritual and religious factors integrated into group psychotherapies and, furthermore, to highlight important issues concerning spiritual and religious factors that research has left unresolved.

## 2. Theoretical Perspectives

Several studies have indicated that people's spirituality and/or religious faith and practice increase, when experiencing personal crisis due to illness or other circumstances [15–17]. Studies have also revealed how spirituality and religion as a meaning-system, distinguished from other meaning-systems, play a significant role for people in crisis [18–21]. The meaning-function of spirituality and religion for people in crisis may be superior compared to other meaning-making resources because spirituality and religion entail belief in a higher principle or force that goes beyond human life and that may provide help and comfort during crisis. The spiritual and religious meaning-function offers meaning in all aspects of human life from birth to death and particularly in a believed afterlife [18, 19]. However, even among spiritual and religious people a significant variance within the importance of spirituality and religion as a meaning-system exists. For some, spirituality and religion are at the center of their lives, and, for others, spirituality and religion play a minor role in their psychological well-being [3]. Therefore, the importance of spirituality and religion to the individual can be expected to influence the spiritually and religiously integrated psychotherapy as clients' motivation for therapy and faith in the therapy are crucial factors for determining the outcome of the therapy [22]. We will elaborate on this by presenting the common factors models after defining spirituality and religion as it is applied in the paper.

It is challenging to define spirituality and religion and to differentiate between the two concepts [23]. However, definitions and operationalization of these concepts in empirical studies will affect the focus and the outcomes of the study, and insufficiently defined concepts will be a source of error.

There are different approaches to studying spirituality and religion, and Zinnbauer et al. [24] divide these into traditional and modern approaches. The traditional approaches to studying spirituality and religion view religion as a broadband construct, where spirituality is not explicitly differentiated from religion but much rather is integrated to it and characterized as *lived religion *or *piety *[25]. Within traditional approaches personal religiosity is emphasized, and religion can be both a positive and a negative construct. The modern approaches, however, view religion as a narrowly defined construct, polarized from spirituality. The modern approaches emphasize religion as external, instrumental, and “bad”, whereas spirituality is personal, relational, and “good” [26]. Zinnbauer et al. [24] and Pargament [26] criticize the traditional approaches for not distinguishing between spirituality and religion and the modern approaches for polarizing the two concepts. Pargament [26] critically discusses the problems with this polarization of spirituality and religion.

Pargament forwards three main critiques. The first critique concerns the tension between the two concepts, which many theorists emphasize but which most believers do not experience. Surveys in the United States conducted by Zinnbauer et al. [27] have shown that when forced to choose 74% label themselves as both religious and spiritual, 19% are spiritual but not religious, 4% are religious but not spiritual, and 3% are neither religious nor spiritual. A cross-cultural study conducted by Keller et al. [28] indicated that the same pattern can be observed in Europe. Thus, the distinction has been characterized rather as a humanistic depreciation of religion more prevalent in academia than in the world of believers [29].

Pargament's second critique concerns the decontextualization of spirituality. By their definition of spirituality, most theoreticians assume that the spiritual dimension of life unfolds in a vacuum. Pargament argues that the spirituality of the individual arises, develops, and unfolds in a larger religious context, even if that context has been rejected. Many researchers agree. Thus, for instance, Moberg [30] is critical of the possibility of evaluating spirituality per se and calls researchers to be context-aware and implement measurement instruments targeted at the particularities of the religious group of people under scrutiny.

Pargament's third critique concerns romanticizing spirituality as only positive, personal, and linked to the best in human nature. Confronting such a notion, Pargament emphasizes that the spiritual dimension of life can be both constructive and destructive [9]. In the same vein, Koenig [31] argues that this positive understanding of spirituality has affected the instruments used to measure spirituality; measures of spirituality are contaminated with positive psychological traits or human experiences. Spirituality will always correlate with mental health if positive mental health and human values become a definition for spirituality. Spirituality, gauged by good mental health measurements, will always be tautologically correlated with good mental health [31].

The importance of clear definitions and operationalization of these concepts is also apparent in empirical studies and clinical praxis. Obscure definitions create uncertainty about what is actually being studied and integrated into psychotherapy. The problem of tautology will affect the outcomes and can become a source of error of a study. Furthermore, without clear definitions psychologists and therapists in the clinical praxis are without guidelines when they seek to integrate spirituality and religiosity.

For this study, we applied the definition for religion and spirituality propounded by Pargament. He defines religion as *the search for significance in ways related to the sacred*, and spirituality as *the search for the sacred*. These definitions take into account the critiques proposed above. These definitions are dynamic because they incorporate the motivating force within all people towards spirituality and they take into account both the positive and negative aspects of spirituality. Furthermore, Pargament believes that the most critical function of religion is spiritual in nature. Despite the many purposes of religion, its most essential function is the desire to form a relationship with something or someone considered sacred.

In the present paper, the differentiation between traditional and modern approaches, Pargament's three points of critique of the modern approaches and Koenig's critique of tautological measurements, will be used to evaluate the definitions used in the studies and the spiritual or religious outcomes presented in the studies.

In order to critically evaluate the effect of integrating spirituality and/or religiosity in group psychotherapies, we found it necessary to also take into account other psychological factors, such as the common factors [22] of psychotherapy, which could have affected the outcome of the interventions.

The medical model has dominated research in psychotherapy. The medical model emphasizes that the main purpose of research in psychotherapy is to examine the effect of specific therapies on specific mental illnesses [32]. The medical model assumes that there is a psychological explanation for the patient's mental disorder, and that there is a mechanism of change consistent with this theoretical explanation. The mechanism of change then suggests a particular therapeutic action, and this action is solely responsible for the benefits of psychotherapy [33].

As a response to the medical model, Duncan et al. [22] propounded the *common factors models*. The common factors models emphasize the collaborative work of the therapist. They focus on the therapist, the client, the transaction between them, and the structure of the treatment that is offered [33]. Hubble et al. [34] divide the common factors in four elements. (1) *Client and extratherapeutic factors* encompass all that affect improvement independent of treatment, for example, clients' readiness for change, strengths, resources, level of functioning before treatment, social support network, socioeconomic status, personal motivations, and life events. (2) *Models and techniques* encompass the clients' and therapists' faith in the restorative power and credibility of the therapy. (3) *Therapist factors* concern the effectiveness of the person of the therapist. Evidence suggests that effective therapists use the common factors to achieve better outcome. (4) *Therapeutic relationship or alliance* concerns the partnership between the client and therapist to achieve the client' goals. A positive alliance is one of the best predictors of outcome [34]. Contrary to the medical model, the common factors models assume the mechanism of change to be complex, and therefore a particular therapeutic action cannot be solely responsible for the outcome of psychotherapy.

In the present review, the medical model and the common factors model with the four elements presented by Hubble et al. [34] will be used to evaluate and discuss the outcomes, the definitions, and the spiritual or religious factors of the group psychotherapies.

## 3. Aim

To systematically review the research literature to answer the following questions.

1. How are spirituality and religiosity defined?
2. How are spiritual and religious factors characterized and integrated into group psychotherapy?
3. How is the outcome of the group psychotherapies measured and what are the results?

## 4. Method

This study was designed as a systematic literature review.

### 4.1. Search Strategies

In the search process for the literature on spirituality and religion in group psychotherapies, two overall search strategies were used: (1) a combination of “brief” and “building block” search strategies (searching databases) and (2) a “citation pearl growing strategy” (systematic reviewing reference lists for the further relevant literature) [35]. The first author performed the search for the literature, which was concluded in April 2013. Two databases were searched, PsycINFO and PubMed, because a wide range of potentially relevant journals for psychology and healthcare are indexed in these databases. Different “brief” and “building blocks” search strategies were explored in order to obtain as many references as possible and create similar searches in the two databases. The controlled headings in PsycINFO (Index terms) included “Religion,” “Religiosity,” “Religious Beliefs,” and “Spirituality,” and a brief search of these four Index terms combined with the Index terms “group psychotherapy” and “Group Intervention” identified 95 references. PubMed's controlled headings (MeSH terms) “Religion,” “beliefs, religious,” and “spirituality” were combined with the MeSH term “group psychotherapy,” and the search identified 221 references. The software program EndNote was used to handle the references. Seven references overlapped, and the total of 309 retrieved references from the database search were examined by titles and abstracts to see if they met the inclusion criteria. Ninety-nine articles were considered eligible for full-text examination, which indicates a relatively high level of “precision” for the database search [35]. Further, the reference lists of the 99 full-text articles were examined as a part of the “citation pearl growing strategy” [35]. Only three additional articles were found as a part of the “citation pearl growing strategy”, which indicated a high level of “recall” [35]. The 102 articles were full-text examined to meet the exclusion criteria for the study.

### 4.2. Inclusion Criterion

Articles reporting English and Scandinavian language empirical studies on spiritually or religiously integrated psychological group intervention.

### 4.3. Exclusion Criteria

The exclusion criteria for the review were as follows.

- Studies on interventions where the spiritual or religious element is only a minor part of a cultural or social understanding.
- Studies on an integration of specific “spiritual” techniques into intervention (e.g., yoga, meditation, and forgiveness) where the overall intervention is not informed by spiritual or religious considerations.
- Studies where the focus is on a specific type of intervention (e.g., art-based or psychosocial) and the spiritual element is secondary.
- Studies on psychoeducational group interventions.
- Studies on couples and family interventions.
- Studies on existential and meaning-centered group interventions that did not specifically include religious or spiritual elements.

### 4.4. Quality Assessment

In total, 10 articles met the inclusion and exclusion criteria for the review. The first author evaluated the studies based on checklists from standardized assessment tools. The intention of using checklists was to quality assess the methodological rigor of the ten studies by the objective of the type of study presented and to omit methodological vague studies. Qualitative studies (*n* = 2) were subject to quality assessment using the Critical Appraisal Skills Program [36]. Quantitative studies (*n* = 8) were subject to a checklist developed by Regan et al. [37]. See Table 1 for quality assessment checklists.

**Figure 1:**
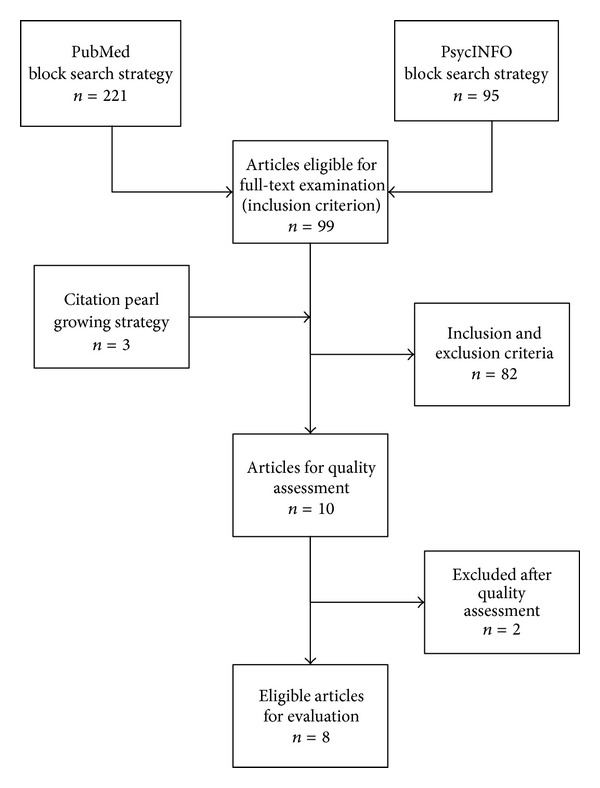
Search strategy and exclusions.

In the quality assessment three types of evaluation were used: 0 for not reported item, 1 for insufficient reported item (e.g., implied information), and 2 for sufficient reported item (e.g., explicit information). The quality assessment of the papers led to the exclusion of two studies [38, 39]. See Figure 1 for search strategy and exclusions.

### 4.5. Evaluation of Interventions

In order to evaluate the spiritually or religiously integrated group psychotherapies three specific questions were added to the review process.

1. How were spirituality or religion defined for the group psychotherapy?
2. How were spiritual or religious factors integrated into the group psychotherapy?
3. What was the outcome of the spiritually or religiously integrated group psychotherapy?

The evaluation is presented in Table 2.

## 5. Findings

The eight articles in the sample were considered methodologically transparent and therefore eligible for the review. There were general weaknesses in all studies, which included a lack of discussions on ethical issues, and most of the quantitative studies only vaguely addressed issues on probability sampling and response rates. However, the remaining eight articles scored high on methods, measures, analysis, findings, and the value of the research. This positively impacts interpretation of their findings. See Table 2 for assessment scores.

In the following sections, after a brief general description of the included studies, we will review the studies in terms of (1) definitions of spirituality and religion, (2) description of the spiritual and religious factors in the studies, and (3) outcome of group therapies.

### 5.1. Description of Group Psychotherapies

Several types of group psychotherapies were presented in the eight studies. The duration of the sessions varied from 45 minutes to two hours. Four of the group psychotherapies presented were time-limited interventions with six to fourteen sessions. Two studies reported on group psychotherapies without limits to numbers of sessions. One study did not report duration or number of sessions [40]. One study reported an intensive treatment model with twelve weeks of daily treatment [41].

Seven of the group psychotherapies were aimed at specific groups of patients: adults with major mental illness [42]; HIV-positive drug users [40]; HIV patients [43]; perfectionism among Mormon college students [44]; Buddhist diabetes patients with depressive symptoms [45]; patients recovering from schizophrenia [46]; women with primary breast cancer [47]. Only Austad and Folleso [41] reported on a group-based treatment for patients, whose religious and existential experiences were an important part of their mental illness.

Three group psychotherapies aimed their interventions at persons with a preceding interest in spirituality or religion: *Vita-prosjektet* [41] was only for people with an outlined interest in religious issues; the Buddhists group therapy [45] only accepted Buddhists; the Mormon perfectionism group [44] were specifically designed for Mormons; the spirituality-oriented group intervention for HIV-positive adults [43] were only for HIV patients with a specific interest in spirituality. The other four group interventions were aimed at specific patient groups, which did not necessarily have a preceding interest in spirituality or religiosity.

### 5.2. Definitions of Spirituality and Religion

Definitions of spirituality or religion were entirely absent in three of the eight studies [40, 41, 44], and the lack of any conceptualizations caused uncertainty about how spiritual or religious factors were integrated into the group psychotherapies presented.

O'Rourke [42] used the modern approach of defining these two concepts (see Pargament's distinction above). Religion was defined as the individual's religious affiliation or denominational background, whereas spirituality concerned the individual's values, relationships, and perceptions of the sacred; religion was defined as an institutional construct, whereas spirituality was concerned about the individual and her or his sacred experiences. However, the group therapy O'Rourke presented solely addressed spiritual issues. He defined spirituality as a solely individual and personal construct and did not use his definition for religion in the study.

The study by Rungreangkulkij et al. [45] used a traditional approach to defining (see Pargament's distinction above), where religion is the broadband construct, and spirituality is not explicitly differentiated from religion [24]. Rungreangkulkij defined Buddhism where spirituality was a concurrent and integrated part of the Buddhist religion.

The studies by Revheim et al. [46], Garlick et al. [47], and Tarakeshwar et al. [43] all used modern approaches to defining, and they romanticized spirituality as only positive, personal, and linked to the best in human nature [26]. Spirituality was defined as personal beliefs, practices, and values and these related to meaning, purpose, and renewed engagement with life. Spirituality could also stem from a particular denomination normally associated with religion or faith in a higher purpose or power.

Only the study by Tarakeshwar et al. [43] defined spirituality as possible also being a relationship with God or a higher power, and, as the only study using a modern approach, they understood spirituality as a construct with both positive and negative aspects. The explicit theoretical and empirical foundation for the group intervention was Pargament's concepts of religion and religious coping [15]. Tarakeshwar et al. [43] emphasized that each patient should define their individual spirituality in the first group session. Thereby, spirituality was a solely individual and personal construct. They also emphasized that studies have shown that individuals with HIV are more likely to define themselves as being spiritual rather than religious and they therefore focused on spirituality and omitted religion from the group therapy. This contradicts Pargament's [26] first critique about patients not making the distinction between religion and spirituality, and it is not coherent with the definition and understanding of religious coping presented by Pargament [15].

*Summing up:* Definitions of spirituality and religion in the eight studies were characterized by a strong emphasis on spirituality whilst religion was mostly omitted. Three studies did not report any conceptualization of spirituality and religion at all. Spirituality was individually defined with broad positive constructs. In the same vein, some studies purposely avoided clear definitions, as they wanted clients to fill the concepts with their own individual meaning.

### 5.3. The Spiritual and Religious Factors

The purpose of “the spiritual issues group for adults with mental illness” [42] was to offer the clients a safe place to explore their spiritual issues. The spiritual factor in this group therapy would be a spiritual safe place. However, due to the individually and solely positive definition of spirituality for the intervention, a spiritual safe place could be almost everything that felt “good” to the patients within the group therapy. Thereby, the spiritual factor became unclear, and it could be questioned if the group therapy was separated from other types of group psychotherapies without an integration of spirituality.

Margolin et al. [40] presented no definitions for spirituality or religion for the spiritual self-schema therapy. Each individual should create, strengthen, and activate an individually meaningful spiritual self-schema. The spiritual self-schema could be the spiritual factor in this group therapy. However, the spiritual factor became obfuscated because the spiritual self-schema had to be created by the individual for individual meaning. Thereby, the spiritual factor could be anything personal and meaningful taking place in the group therapy, and the outcome of the group therapy may not be directly connected to the spiritual factor.

Richards and Owen [44] had implemented a group intervention developed by King [48] and added a religious-spiritual component. They had not defined spirituality or religion. Despite the lack of definitions, religious imagery and discussions of religious bibliotherapy articles and the relationship between religious beliefs and perfectionism were integrated into the group therapy. However, the spiritual/religious factor of the group therapy was difficult to assess, because the group therapy addressed the Mormons' religious beliefs but without defining those religious beliefs. The intervention was concerned about using the above-mentioned “religious tools” to address religious beliefs that exacerbated perfectionisms. But, because the religious beliefs were undefined, it remained unclear if the “religious tools” addressed them. Furthermore, it was questionable if their self-defeating perfectionism group for Mormons could be separated from other self-defeating perfectionism groups.

Rungreangkulkij et al. [45] defined Buddhism for the therapy as the three universal laws of Buddhism and integrated the definition; they presented a religious definition and created a religious intervention. The purpose of the group therapy was for the participants to live as good Buddhists. The religious factor was easily identifiable because the whole intervention was religious. The entire Buddhist group intervention was the religious factor.

The studies by Revheim et al. [46] and Garlick et al. [47] defined spirituality as a solely positive and personal construct. The foci were primary on a personal sense of meaning. The spiritual factors at work in their group therapies were unclear and difficult to assess. It was unclear if the interventions were “spiritual” or “positive” because spirituality was solely something positive in their definitions. Thereby, the spiritual factors in the group therapies could be anything the patient experiences as positive within the context of the group therapy. This questioned if these group psychotherapies were separated from other types of group psychotherapies without integration of spirituality.

“Vita-prosjektet” presented by Austad and Folleso [41] was based on object-relational theory. The focus was on the patients' God representations and how these influenced the lives and psychic function of the patients. Neither spirituality nor religion was defined for this study. However, the integration of spirituality and religiosity through God representations was theoretically and empirically understood and defined. The spiritual/religious factor in this group therapy was God representations. They presented a clear delimited spiritual/religious factor for the group therapy.

Tarakeshwar et al. [43] presented a detailed description of the content for the spiritual coping group intervention for HIV patients. Positive spiritual coping was the focus of the group therapy, and the patients should reflect on how spirituality helped or hindered coping with HIV. Tarakeshwar et al. focused on spirituality and omitted religion, and they emphasized an individual self-definition for spirituality. However, examining the group intervention the underlying theory became apparent. The theoretical and empirical foundation for the group intervention was Pargament's concepts of religion and religious coping [15]. Despite the fact that Pargament's theory is on religious coping and Tarakeshwar et al. incorporate their theory into a solely spiritual intervention rooted in clients' self-definitions of spirituality, the purpose of the group therapy was for the participants to increase their positive spiritual coping. The spiritual factor was therefore easily identifiable because the whole group therapy was spiritual.

Summing up, the descriptions of spiritual or religious factors were unclear in five of the studies. The outcome of the group interventions may or may not be directly connected to the spiritual or religious factors at work in the group therapies presented, and it remains unclear whether these group therapies are separated from other types of group therapies without an integration of spirituality or religiosity. Only the studies by Rungreangkulkij et al. [45], Tarakeshwar et al. [43], and Austad and Folleso [41] had integrated spiritual or religious factors in the group interventions that could be expected to be directly related to the outcome of the intervention. Based on the clarity and delimitations of the spiritual/religious factors in these three group therapies, it was possible to distinguish them from other types of group therapies without an integration of spiritual or religious factors.

### 5.4. Outcome of the Group Therapies

O'Rourke [42] reported on qualitative findings from a spiritual issues group with 12 adults with mental illness. He presented different themes that had emerged from the data. The data of the study suggested that addressing spiritual issues into group psychotherapy facilitated integration of the individual's spirituality with all other dimensions of one's personality. However, O'Rourke's study had the weakness that it did not account for how the researchers'/interpreters' preconceptions influenced the data and findings of the study.

Margolin et al. [40] used a controlled pretest-posttest design to study an eight-week spirituality focused group therapy. Forty HIV-positive drug users received acupuncture treatment and “the last” 15 of them also received “spiritual self-schema therapy”. Measurements included depression (BDI), anxiety (STAI), drug urine tests, and general ratings of the effect of acupuncture. Both groups reported reductions in depression (BDI) and anxiety (STAI). The follow-up period was not reported. The spiritual self-schema group reported greater reductions than the “acupuncture only” group, but the intergroup differences were not significant. Urine tests indicated that the spiritual self-schema group was abstinent from heroin and cocaine for significant more weeks than the “acupuncture only” group.

Richards and Owen [44] used a pretest-posttest design, where they completed the outcome measures eight weeks after ending group treatment. Fifteen Mormons received the group intervention for self-defeating perfectionism. Measurements included depression (BDI), perfectionism (PS), self-esteem (CSE), and the religious and existential well-being subscales of SWBS. The participants scored significantly lower on depression (BDI) and perfectionism (PS) and higher on self-esteem (CSE) and existential well-being (subscale of SWBS) at the conclusion of the group. There was no significant increase of religious well-being (subscale of SWBS), which indicated that the effects on depression and perfectionism were not caused by religious well-being. Moreover, the measures included the same or similar items creating self-enforcing, tautological effects.

Rungreangkulkij et al. [45] presented a pretest-posttest design with a matched control group of 32 patients and 32 patients attending a “Buddhist group therapy.” The measurement used was change in depression symptoms (PHQ-9). It was administered before intervention and six months after intervention. The continuous PHQ-9 scores (ranging from 0 to 27) indicated that both groups were less depressed: the Buddhist group scored 11.8 (pretest) and 1.0 (posttest) and the control group 11.5 (pretest) and 5.9 (posttest), but no significance tests were made of these intergroup differences. In a subsequent intention to treat analysis, the PHQ-9 were categorized as normal (scores < 7) and depression (≥7) and it indicated that participants in the intervention group had a significantly greater opportunity (6.6 times) to turn to normal compared to the control group.

Revheim et al. [46] designed a follow-up study, where they compared group attendees (*n* = 20) with a matched control group (*n* = 20) after ending intervention. Measurements included spiritual status (SSQ), self-efficacy (SES), quality of life (QOL), hopefulness (HHI), and religious/demographic profiles. They found that the group-attendees-spirituality status (SSQ) was significantly correlated with self-efficacy (SES) and hope (HHI), and the group attendees had a significantly higher spiritual status and hopefulness score than nonattendees. However, they used instruments where constructs were measured with same or similar items (e.g., SSQ measuring same or similar items as HHI), which again can create tautological effects, and there was a relatively limited number of significant results considering the extensive use of measurements. 

Garlick et al. [47] used a pretest-posttest study design, where they administered measurement instruments in three different time periods: a baseline assessment, postintervention assessment within a week after completion of intervention, and follow-up assessment four weeks later. Instruments were selected to measure quality of life (FACT-B), mood disturbance (POMS), posttraumatic growth (PTGI), and spiritual well-being (FACIT-Sp-Ex). They reported on 24 women with primary breast cancer completing a “psychospiritual integrative therapy” and 20 women completed the follow-up instruments. Participants improved psychological and physical well-being (POMS and FACT-B), spiritual well-being (FACIT-Sp-Ex), and posttraumatic growth (PTGI). Significant effects for time with significant improvements were found between pretest and posttest and between pretest and follow-up. However, the follow-up period was short for determining lasting changes among the participants, and they also administered tautological assessment instruments.

Austad and Folleso [41] used a pretest-posttest design. Measurements included general symptoms (SCL-90), depression (BDI), and interpersonal problems (IIP). The 23 patients completed the intervention, and they all attained a significant reduction in symptoms. The average score for general symptoms (SCL-90) was reduced to 0.7 from 1.2, and the average score for depression (BDI) was reduced to 8.8 from 19.8. Only two patients fulfilled the criteria for interpersonal problems (IIP) preintervention, but these also displayed a significant positive change. The period between pretest and posttest was not reported.

Tarakeshwar et al. [43] evaluated the effectiveness of a spiritual coping group intervention for 13 adults living with HIV/AIDS using a pretest-posttest design. They administered assessment instruments on religious beliefs and practices (selected subscales of BMMRS), psychological distress (CES-D), and demographic characteristics before intervention and three weeks after intervention. They found that after intervention participants experienced significantly higher religiosity (BMMRS), lower use of negative spiritual coping (BMMRS), and lower depression (CES-D). The participants also experienced more use of positive spiritual coping (BMMRS) but not significantly more. However, the follow-up period was relatively short, and there were a relatively limited number of significant findings relative to the number of variables measured.

All eight studies reported some positive outcomes of the religiously or spiritually integrated group psychotherapies. However, none of the studies used randomized designs, samples were relatively small, the instruments used for measuring outcomes in half of the studies to some degree tautologically measured the same construct, and none of the studies tried to minimize the Hawthorne effect. Despite the reports of positive outcomes, the study designs presented in the eight studies were not robust, and there is no solid evidence for positive or direct outcomes of integrating religious and spiritual factors into group therapy. However, absence of evidence is not evidence of absence and further studies with more robust designs are needed in this undeveloped field of research.

## 6. Discussion

For some people, spirituality and religion are at the center of their lives, and, for others, spirituality and religion play a minor role in their psychological well-being [3]. The variance and importance of spirituality or religiosity in patients can be expected to influence both the spiritual and/or religious factors at work in the group psychotherapies as well as their outcome. Only the group interventions presented by Austad and Folleso [41], Tarakeshwar et al. [43], Richards and Owen [44], and Rungreangkulkij et al. [45] proposed a group therapy for patients with a specific interest in religion and spirituality. It is surprising that the remaining four studies did not voice any explicit concern for this, as the motivation of the clients before entering psychotherapy is considered an *extratherapeutic factor* which can be crucial to psychotherapy [34].

All eight studies applied the medical model to measure the effect of the total intervention, and none of them addressed the common factors at work. This is likewise surprising, as integration of religion and spirituality into group psychotherapy can be said to be *model or techniques factors* that induce positive expectations and assist the clients' participation in the therapy [34]. Furthermore, the evaluation showed that for most of the studies the spiritual or religious factors integrated into the group therapies could not safely be directly connected to the outcome of the group therapies. If the studies had applied a common factors model instead of the medical model for measuring the outcome of the group therapies, it could have revealed clearer delimitations between these eight spiritually and religiously integrated group psychotherapy and group psychotherapies without integration of spiritual or religious factors.

The outcomes of the eight group therapies remained questionable because the definitions and conscious integration of spiritual or religious factors in the group therapies—for the majority of the studies—were unclearly described and not necessarily connected to the outcome of the studies and also due to their use of weak study designs, limited samples, and tautological assessment tools.

The lack of clear identification of the spiritual and religious factors and their relations to the outcome might suggest that the outcome of the studies were caused by *common factors* [22]. The four elements of common factors presented by Hubble et al. [34], client and extratherapeutic factors, models and techniques, therapist factors, and therapeutic relationship or alliance, could all have been present in all the group therapies, and they could all suffice directly or indirectly in causing the outcome of the studies.

Finally, several of the studies presented modern definitions for spirituality and religion, where spirituality is a solely positive and personal construct [26]. Thereby, spiritual factors became anything the clients might experience positive within the group therapy. For these studies, the spiritual factors were questionable because the concept of spirituality remained unclear.

Considering limitations of the present systematic review, it should be noted that only one researcher (the first author) conducted the literature search, whereas all three authors conducted the complete evaluation. However, the search strategies have been described in detail, ensuring transparency, and the evaluations were standardized and made on the basis of the structured evaluation tools.

## 7. Conclusion

Clear and delimited conceptualization of spiritual/religious factors is crucial in order to be able to conclude the direct influences of spiritual/religious factors on outcomes. The studies by Rungreangkulkij et al. [45], Tarakeshwar et al. [43], and Austad and Folleso [41] had successfully integrated spiritual/religious factors into group psychotherapy and had delimited the spiritual/religious factors of the group interventions, so these became clear and specific. Despite limitations of study designs and a need for more rigorous study methods, the spiritual/religious factors of these studies were considered directly connected to the outcome of the group psychotherapies. And the spiritually or religiously integrated group psychotherapies presented differentiated from other types of group psychotherapies without spiritual or religious factors. It seemed that romanticizing spirituality, as a solely personal and positive construct, would obfuscate the spiritual factors of the group therapy. However, a complete lack of definitions for religion and spirituality would only be a problem if the religious and spiritual factors also remained undefined and unclear. Furthermore, these studies had addressed groups of patients with an outlined interest in religious and spiritual issues, and this seemed to call for patients' motivation and common factors, which affected group therapy and outcome positively.

The above evaluation has implications for spiritually or religiously integrated group psychotherapy. Based on this systematic review study, it would seem that clear and delimited conceptualizations of the spiritual or religious factors form the basis for spiritually or religiously integrated group psychotherapy. Furthermore, to aim the spiritually or religiously integrated group psychotherapy at people with specific interests in the spirituality and religiosity seems to increase patients' motivation for therapy.

Furthermore, the evaluation has implications for research on spiritually and religiously integrated group therapy. It is an underresearched area of inquiry, and the articles of the present review can all be said to have used weak study designs. This new area of research thus calls for more studies and for robust randomized study designs. Especially, it would be necessary with studies having a control group that did not have the spiritual factors. This would provide the best comparison and allow one to test for the effects of the spiritual factors. For the area to provide solid evidence of any effect of integrating religion and spirituality into group therapy, a consensus within the field of religion, spirituality, and health about measures for spirituality and religion that are not contaminated with items for mental health is warranted. 

## Figures and Tables

**Table 1:** Quality assessment checklists.

Qualitative studies	Quantitative studies
(1) Are the aims clearly stated?	(1) Target population: clear inclusion and exclusion criteria?
(2) Is a qualitative methodology appropriate?	(2) Was probability sampling used?
(3) Was the research design appropriate to the research aims?	(3) Did respondents' characteristics match the target population; that is, was the response rate ≥80%?
(4) Was the recruitment strategy appropriate to the research aims?	(4) Were data collection methods standardised?
(5) Were data collected in a way that addressed the research issue?	(5) Was the measure used valid?
(6) Has the researcher-participant relationship been adequately considered?	(6) Was the measure used reliable?
(7) Have ethical issues been considered?	(7) Have ethical issues been considered?
(8) Was the data analysis sufficiently rigorous?	(8) Was the data analysis sufficiently rigorous?
(9) Is there a clear statement of findings?	(9) Is there a clear statement of findings?
(10) How valuable is the research?	(10) How valuable is the research?

Regan et al. [37].

**Table 2:** Evaluations and quality assessment scores.

Authors, year, and country	Study design (*n* = ?)	Measures	Effect of the group therapy	Type of group therapy	Definitions	Religious/spiritual factors	Quality assessment scores									
							1	2	3	4	5	6	7	8	9	10
O'Rourke (1996) [42] USA	Qualitative, exploratory design: (*n* = 12)	Audiotaped and transcribed the therapy sessions.	Addressing spiritual issues in group psychotherapy greatly facilitate an integration of spirituality with all other dimensions of the individual's personality.	Spiritual issues group (psychodynamically oriented) for adults with major mental illness.	*Religion*: the individual's religious affiliation or denominational background. *Spirituality*: the individual's ultimate values, relationship with others, and perception of the sacred which may be expressed within or outside the context of religious tradition.	Creating a spiritual safe place for raising and exploring spiritual issues.	2	2	1	2	1	1	0	2	2	2

Goodman and Manierre (2008) [39] USA	Qualitative						0	0	1	0	1	0	0	1	1	1

Margolin et al. (2005) [40] USA	Quantitative pretest-posttest design: acupuncture treatment and 3-S therapy. (*n* = 15)	Drug use: urine samples, depression: BDI, anxiety: STAI.	Patients were abstinent significantly longer. Reductions in depression and anxiety.	Spiritual self-schema therapy (cognitive-behavioral and Buddhist) for treatment of HIV-positive drug users.	Spirituality or religion is not defined.	Create, strengthen, and make the “spiritual self-schema” (3-S) more accessible for activation.	2	2	1	2	2	2	0	2	2	2

Richards and Owen (1993) [44] USA	Quantitative, pretest-posttest design. (*n* = 15)	Depression: BDI, perfectionism: PS, self-esteem: CSE. Religious/spiritual well-being: SWBS.	Participants scored low on depression and perfectionism, and high on self-esteem and existential well-being.	Group counseling (cognitive methods) intervention for self-defeating perfectionism with devout Mormon clients.	Spirituality or religion is not defined.	Address religious beliefs that exacerbate perfectionistic tendencies and make these tendencies more difficult to overcome.	1	0	0	2	2	2	0	2	2	2

Rungreangkulkij et al. (2011) [45] Thailand	Quantitative, pretest-posttest design with matched control group: (*n* = 32)	Depression: PHQ-9	6-month followup: 65.5% of control group and 100% of Buddhist group returned to normal.	A Buddhist group therapy for diabetes patients with depressive symptoms.	Buddhistic principles: the three universal laws: (1) impermanence, (2) suffering, and (3) selflessness (no self).	Creating insights about cravings and being able to realize the law of impermanence and nonself.	2	2	1	2	2	2	0	2	2	2

Revheim et al. (2010) [46] USA	Quantitative, follow-up design with matched control group. (*n* = 20)	Spirituality status: SSQ, self-efficacy: SES, quality of life: QOL, hopefulness: HHI.	Group attendees' had significant higher spirituality status and hope than nonattendees.	“The spirituality matters group” for patients with schizophrenia in the recovery process.	*Spirituality*: personal beliefs and values related to the meaning and purpose of life, which may include faith in a higher purpose or power.	Explore nondenominational religious and spiritual themes designed to facilitate comfort and hope.	2	2	1	2	2	2	0	2	2	2

Garlick et al. (2011) [47] USA	Quantitative, pretest-posttest-follow-up design. (*n* = 24)	Physical well-being: FACT-B, psychological well-being: POMS, posttraumatic growth: PTGI, spiritual well-being: FACIT-Sp-Ex.	Participants improved psychological well-being, physical well-being, spiritual well-being, and posttraumatic growth	A Psychospiritual integrative therapy (PSIT) for women with primary breast cancer.	*Spiritualty*: a variety of practices and beliefs that may or may not stem from a particular denomination. Includes meaning, faith-based, and existential coping components.	Addressing worldviews, life purpose, and life meaning.	2	1	1	2	2	2	1	2	2	2

Austad and Folleso (2003) [41] Norway	Quantitative, pretest-posttest design. (*n* = 23)	General symptoms: SCL-90, depression: BDI, interpersonal problems: IIP.	The average of the patients' general symptoms went from 1.2 to 0.7. The average for depression went from 19.8 to 8.8.	“Vita-prosjektet” for patients who have religious and existential experiences as an important element in their illness.	Spirituality or religion is not defined.	Address God representations.	1	0	0	1	2	2	0	1	2	2

Tarakeshwar et al. (2005) [43] USA	Quantitative, pretest-posttest design. (*n* = 13)	Religious beliefs/practices: selected subscales from BMMRS, psychological distress: CES-D.	Patients reported higher self-rated religiosity, less negative spiritual coping, lower depression, and more positive spiritual coping.	A spiritual coping group intervention for HIV patients.	*Spirituality*: relationship with God/higher power, renewed engagement with life, relationship with family.	Reflect on how spirituality helped or hindered coping with HIV.	1	0	0	2	2	2	0	1	2	2

Jimenez (1993) [38] USA	Quantitative						0	0	1	1	1	1	0	0	1	0

## References

[1] Cobb M, Puchalski CM, Rumbold B (2012). *Oxford Textbook of Spirituality in Healthcare*.

[2] Koenig HG, King DE, Carson VB (2012). *Handbook of Religion and Health*.

[3] Sperry L, Shafranske EP (2005). *Spiritually Oriented Psychotherapy*.

[4] Thygesen LC, Hvidt NC, Juel K, Hoff A, Ross L, Johansen C (2012). The Danish religious societies health study. *International Journal of Epidemiology*.

[5] Cornish MA, Wade NG (2010). Spirituality and religion in group counseling: a literature review with practice guidelines. *Professional Psychology: Research and Practice*.

[6] Coholic D (2005). The helpfulness of spiritually influenced group work in developing self-awareness and self-esteem: a preliminary investigation. *TheScientificWorldJOURNAL*.

[7] Worthington EL, Jr, Kurusu TA, McCollough ME, Sandage SJ (1996). Empirical research on religion and psychotherapeutic processes and outcomes: a 10-year review and research prospectus. *Psychological Bulletin*.

[8] Rye MS, Pan W, Shogren KA, Pargament KI, Yingling DW, Ito M (2005). Can group interventions facilitate forgiveness of an ex-spouse? A randomized clinical trial. *Journal of Consulting and Clinical Psychology*.

[9] Pargament K (2007). *Spiritually Integrated Psychotherapy: Understanding and Addressing the Sacred*.

[10] Richards PS, Bergin AE (1999). *Handbook of Psychotherapy and Religious Diversity*.

[11] Kehoe NC (1998). *Religious-Issues Group Therapy. Spirituality and Religion in Recovery from Mental Illness*.

[12] Schermer VL (2006). Spirituality and group analysis. *Group Analysis*.

[13] Breitbart W (2002). Spirituality and meaning in supportive care: spirituality- and meaning-centered group psychotherapy interventions in advanced cancer. *Supportive Care in Cancer*.

[14] Worthington EL, Sandage SJ (2002). Religion and spirituality. *Psychotherapy Relationships That Work: Therapist Contributions and Responsiveness to Patients*.

[15] Pargament KI (1997). *The Psychology of Religion and Coping: Theory, Research, Practice*.

[16] la Cour P (2008). Existential and religious issues when admitted to hospital in a secular society: patterns of change. *Mental Health, Religion and Culture*.

[17] Koenig HG, Pargament KI, Nielsen J (1998). Religious coping and health status in medically ill hospitalized older adults. *Journal of Nervous and Mental Disease*.

[18] Park CL (2005). Religion as a meaning-making framework in coping with life stress. *Journal of Social Issues*.

[19] Emmons RA (2005). Striving for the sacred: personal goals, life meaning, and religion. *Journal of Social Issues*.

[20] Paloutzian RF (1981). Purpose in life and value changes following conversion. *Journal of Personality and Social Psychology*.

[21] Silberman I (2005). Religion as a meaning system: implications for the new millennium. *Journal of Social Issues*.

[22] Duncan BL, Miller SD, Wampold BE, Hubble MA (2010). *The Heart and Soul of Change. Delivering What Works in Therapy*.

[23] Ahmadi F (2006). *Culture, Religion and Spirituality in Coping*.

[24] Zinnbauer BJ, Pargament KI, Scott AB (1999). The emerging meanings of religiousness and spirituality: problems and prospects. *Journal of Personality*.

[25] Bregman L (2006). Spirituality: a glowing and useful term in search of a meaning. *Omega: Journal of Death and Dying*.

[26] Pargament KI (1999). The psychology of religion and spirituality? Yes and no. *International Journal for the Psychology of Religion*.

[27] Zinnbauer BJ, Pargament KI, Cole B (1997). Religion and spirituality: unfuzzying the fuzzy. *Journal for the Scientific Study of Religion*.

[28] Keller B, Klein C, Swhajor-Biesemann A, Silver CF, Hood R, Streib H (2013). The semantics of “spirituality” and related self-identifications: a comparative study in Germany and the USA. *Archive for the Psychology of Religion*.

[29] Říčan PR (2004). Spirituality: the story of a concept in the psychology of religion. *Archive for the Psychology of Religion*.

[30] Moberg DO (2002). Assessing and measuring spirituality: confronting dilemmas of universal and particular evaluative criteria. *Journal of Adult Development*.

[31] Koenig HG (2008). Concerns about measuring “spirituality” in research. *Journal of Nervous and Mental Disease*.

[32] Hougaard E (2004). *Psykoterapi. Teori og Forskning*.

[33] Wampold BE, Duncan BL, Miller SD, Wampold BE, Hubble MA (2010). The research evidence for the common factors models: a historically situated perspective. *The Heart and Soul of Change Delivering What Works in Therapy*.

[34] Hubble MA, Duncan BL, Miller SD, Wampold BE, Duncan BL, Miller SD, Wampold BE, Hubble MA (2010). Introduction. *The Heart and Soul of Change Delivering What Works in Therapy*.

[35] Harter SP (1986). *Online Information Retrieval. Concepts, Principles and Techniques*.

[36] (CASP) CASP 10 questions to help make sense of qualitative research. http://www.casp-uk.net/wp-content/uploads/2011/11/CASP-Qualitative-Research-Checklist-31.05.13.pdf.

[37] Regan JL, Bhattacharyya S, Kevern P, Rana T (2012). A systematic review of religion and dementia care pathways in black and minority ethnic populations. *Mental Health, Religion & Culture*.

[38] Jimenez MJ (1993). The spiritual healing of post-traumatic stress disorder at the Menlo Park Veteran's Hospital. *Studies in Formative Spirituality*.

[39] Goodman G, Manierre A (2008). Representations of god uncovered in a spirituality group of borderline inpatients. *International Journal of Group Psychotherapy*.

[40] Margolin A, Avants SK, Arnold R (2005). Acupuncture and spirituality-focused group therapy for the treatment of HIV-positive drug users: a preliminary study. *Journal of Psychoactive Drugs*.

[41] Austad A, Folleso GS (2003). Religious and existential issues in psychotherapy. *Tidsskrift for Norsk Psykologforening*.

[42] O’Rourke C (1996). Listening for the sacred: addressing spiritual issues in the group treatment of adults with mental illness. *Smith College Studies in Social Work*.

[43] Tarakeshwar N, Pearce MJ, Sikkema KJ (2005). Development and implementation of a spiritual coping group intervention for adults living with HIV/AIDS: a pilot study. *Mental Health, Religion and Culture*.

[44] Richards PS, Owen L (1993). A religiously oriented group counseling intervention for self-defeating perfectionism: a pilot study. *Counseling & Values*.

[45] Rungreangkulkij S, Wongtakee W, Thongyot S (2011). Buddhist group therapy for diabetes patients with depressive symptoms. *Archives of Psychiatric Nursing*.

[46] Revheim N, Greenberg WM, Citrome L (2010). Spirituality, schizophrenia, and state hospitals: program description and characteristics of self-selected attendees of a spirituality therapeutic group. *Psychiatric Quarterly*.

[47] Garlick M, Wall K, Corwin D, Koopman C (2011). Psycho-spiritual integrative therapy for women with primary breast cancer. *Journal of Clinical Psychology in Medical Settings*.

[48] King MM (1986). *Treatment of Perfectionism*.

